# A rare case of curative endoscopic submucosal dissection of a duodenal gastrinoma revealed by Zollinger-Ellison syndrome

**DOI:** 10.1055/a-2961-1305

**Published:** 2026-10-01

**Authors:** Timothée Wallenhorst, Maria Parmigiani, Mathieu Pioche, Louis-Jean Masgnaux, Thomas Grainville, Mael Pagenault, Jérémie Jacques

**Affiliations:** 1 Department of Endoscopy and Gastroenterology 36684 Rennes University HospitalRennesBrittanyFrance; 2 Translational Medicine and Surgery 60234 Università Cattolica del Sacro Cuore Facoltà di Medicina e ChirurgiaRomeLazioItaly; 3 Department of Gastroenterology and Endoscopy 188700 Fondazione Poliambulanza Istituto OspedalieroBresciaLombardyItaly; 4 Gastroenterology 36609 Hopital Edouard HerriotLyonAuvergne-Rhône-AlpesFrance; 5 Hepatogastroenterology Department 36715 University Hospital Centre of LimogesLimogesFrance

Gastrinomas are rare secreting neuroendocrine tumors (NETs), most commonly arising in the duodenum or pancreas, responsible for Zollinger-Ellison syndrome (ZES). Although ^68^ Ga-DOTATOC positron emission tomography (PET) is the cornerstone of their detection, precise localization of these often millimetric lesions remains challenging, making limited surgical resection difficult to plan. 1​ 2​ 3​ ​4​

To the best of our knowledge, this is the first reported case of curative endoscopic submucosal dissection (ESD) of a duodenal gastrinoma in a patient with ZES ( Video 1 ).

**Video 1** Curative ESD of a duodenal gastrinoma revealing Zollinger-Ellison syndrome, using double clip+rubber band traction and underwater dissection to achieve complete R0 resection. Video URI:


A 50-year-old woman presented with chronic voluminous diarrhea and severe peptic esophagitis. Serum gastrin was elevated 17-fold above the upper limit of normal, and chromogranin A 10-fold. ^68^ Ga-DOTATOC PET-CT identified a single intense duodenal uptake near the pylorus (SUVmax 22.5) with no other pathological foci ( Fig. 1a ). Endoscopy revealed a barely visible, small, flat, yellowish lesion (Paris 0-IIa) in the first portion of the duodenum, confirmed as a NET on biopsy ( Fig. 1b ). Staging was consistent with sporadic duodenal gastrinoma. Following multidisciplinary board discussion, ESD was attempted.

**Fig. 1:**
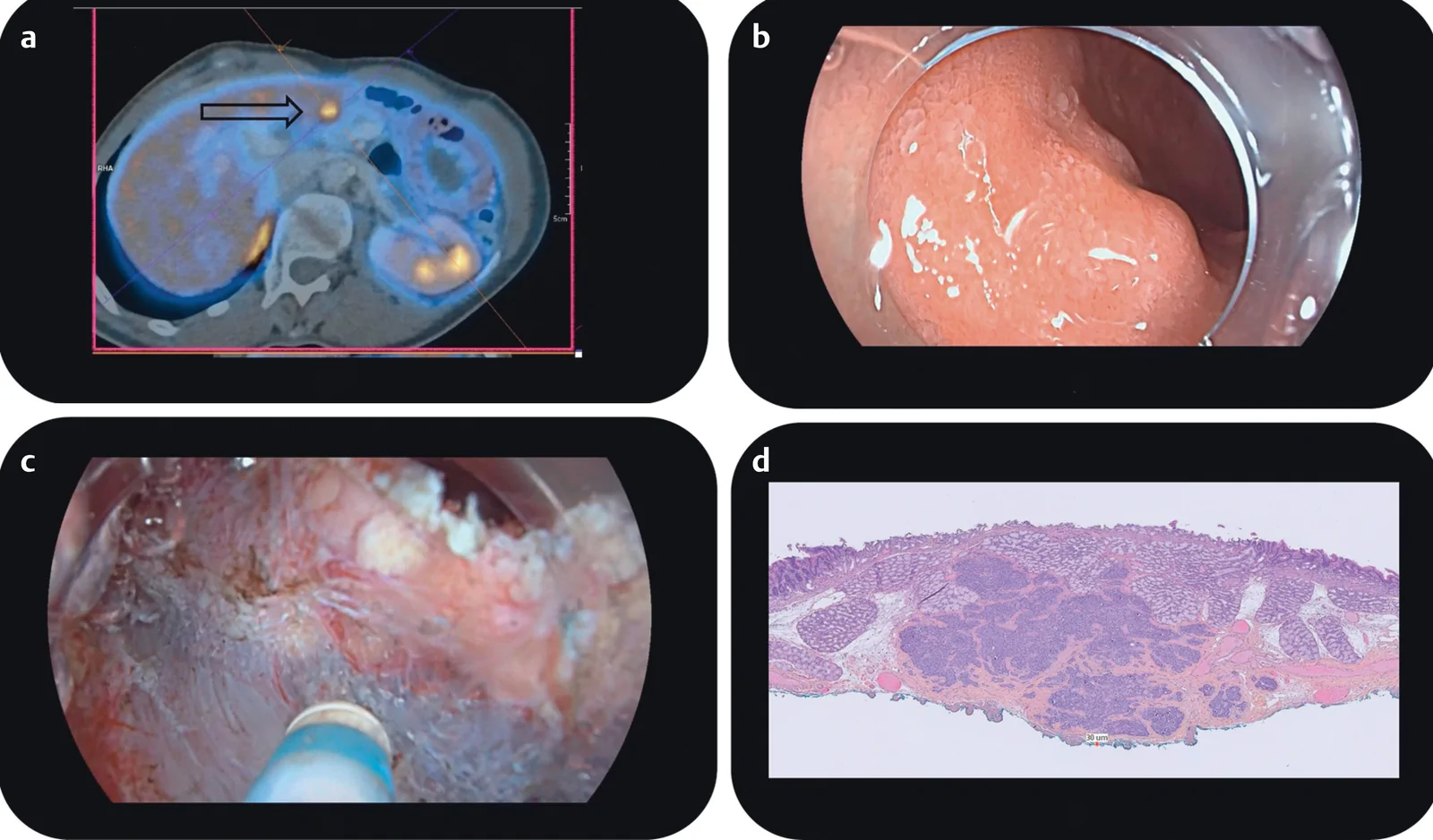
An overview of the case ( **a** ) ^68^ Ga-DOTATOC PET-CT detection of the lesion (arrow). ( **b** ) An endoscopic aspect of the NET. ( **c** ) Submucosal dissection plane close to the muscularis propria. ( **d** ) Histopathological section of the complete resected gastrinoma.

Given the submucosal growth pattern of NETs, dissection was carried close to the muscularis propria using a DualKnifeJ and glycerol+fructose submucosal injection ( Fig. 1c ). To optimize the visualization of the dissection plane, a double clip+rubber band traction strategy combined with underwater dissection in normal saline was employed.

En bloc resection of a 34×32 mm specimen was achieved in 28 minutes with no complications. Histology confirmed a well-differentiated NET (WHO Grade 1, Ki67 1%,<1 mitosis/10 HPF), measuring 3.3 mm, infiltrating the submucosa, with no lymphovascular invasion and complete R0 resection laterally and in depth ( Fig. 1d ).

This case demonstrates that ESD is a feasible and curative option for duodenal gastrinomas in expert centers, provided that advanced exposure strategies—double clip+rubber band traction and underwater dissection—are employed to ensure adequate visualization and safe R0 resection.

Endoscopy_UCTN_Code_CCL_1AB_2AD_3AF

## Declaration of GenAI Use

The authors confirm that no generative artificial intelligence was used in the preparation of this manuscript.
